# Supplementary material for: Niacin Alleviates Dairy Cow Mastitis by Regulating the GPR109A/AMPK/NRF2 Signaling Pathway
Source: Int J Mol Sci. 2020 May 8;21(9):3321. doi: 10.3390/ijms21093321 (PMC7246865; doi:10.3390/ijms21093321)
Supplement: Supplementary file 1 [file ijms-21-03321-s001.pdf]

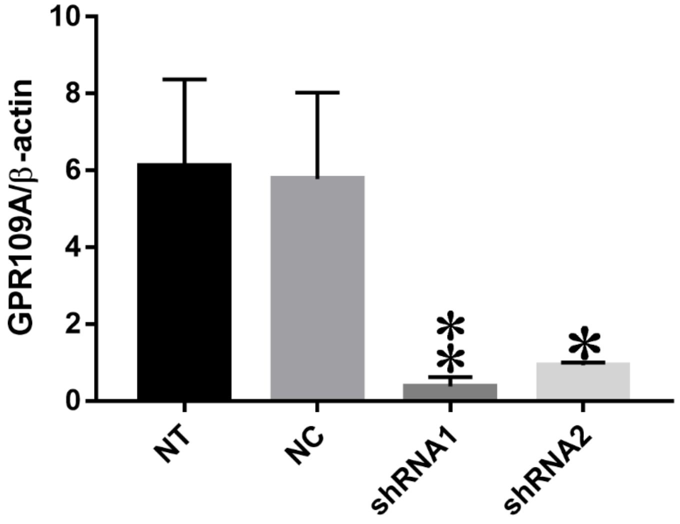

Figure S1. Relative expression of GPR109A. Relative expression of GPR109A after transfection with GPR109A-shRNA1 and GPR109A-shRNA2. The values are presented as the means ± SD ($*p<0.05$ vs NC and $**p<0.01$vs NC).

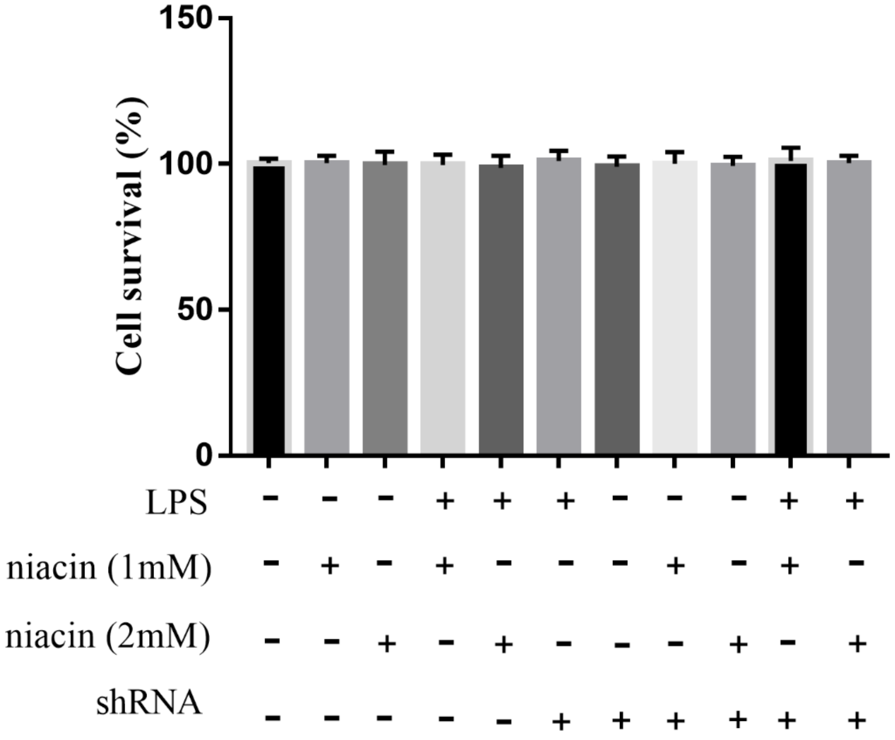

Figure S2. The effect of niacin on the activity of BMECs. The effect of adding niacin, niacin+LPS, LPS, niacin+LPS+shRNA on the BMECs in the identified groups was determined by CCK-8 assay (n=5). The values are presented as the means ± SD. There were no differences between the NT and the other groups.

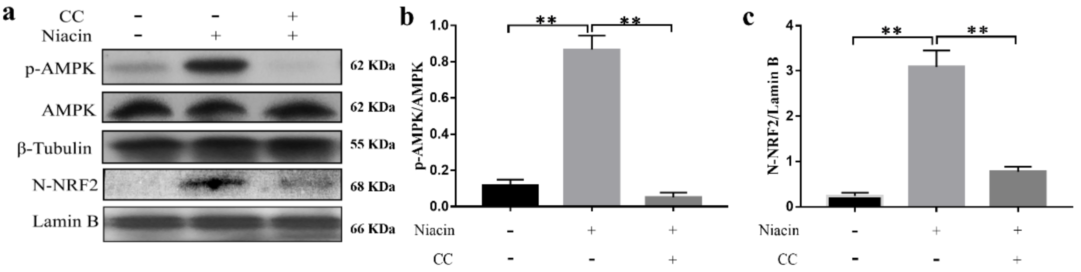

Figure S3. The effects of p-AMPK on NRF-2 nuclear import. The cells from different experimental groups were treated with niacin, CC+niacin or RA+niacin for 24 h, and then, the total protein or RNA was then collected. N-NRF-2 means NRF-2 in the nucleus. The cell lysates were prepared and subjected to western blotting using p-AMPK (a, b) and NRF-2 (a, c) antibodies. Each immunoreactive band was digitized and expressed as a portion of the laminin B or β-tubulin level. Values are presented as the means ± SD (∗p<0.05 and ∗∗p<0.01).

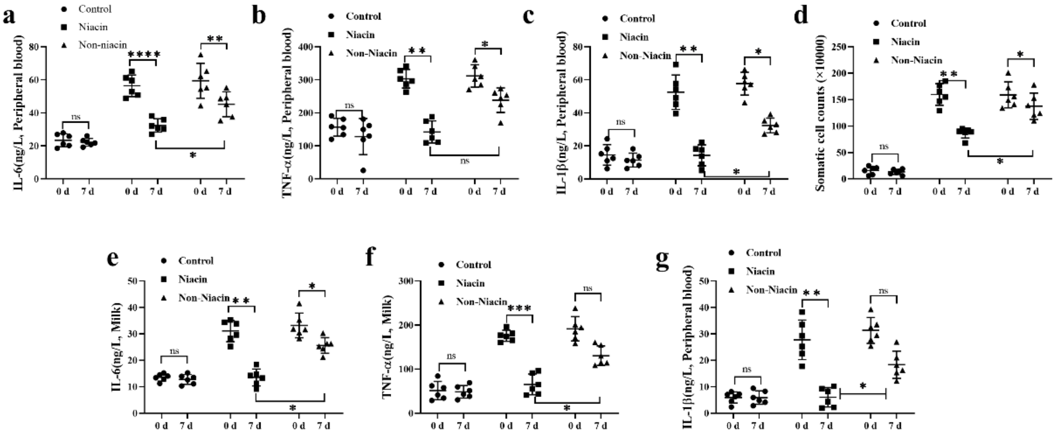

Figure S4. Niacin alleviates dairy cow mastitis by activating GPR109A. Control group was healthy dairy cow; niacin group was mastitis cow fed with niacin (30 g / d, continuous feeding for seven days); non-niacin group was mastitis cow without feeding niacin; (a-c) Contents of IL-6, TNF-α and IL-1β in serum. (d) SCC in milk. (e-g) Contents of IL-6, TNF-α and IL-1β in milk. Values are presented as means ± SD (n=6) ($*p$<0.05, $**p$<0.01 and $***p$<0.001).
